# Knowledge-based Basic Life Support competency and associated factors among healthcare professionals across different hospital levels: a multicenter cross-sectional study

**DOI:** 10.3389/fpubh.2026.1881120

**Published:** 2026-08-20

**Authors:** Pan Pan, YaXiong Zhou, YaoWen Jiang, YiQin Xia, TianYong Han, E. Pan, Yao Chen

**Affiliations:** 1 Department of Emergency Medicine, Disaster Medical Center, West China Hospital, West China School of Medicine, Sichuan University, Chengdu, Sichuan, China; 2 Department of Emergency Medicine, West China Tianfu Hospital, Sichuan University, Chengdu, Sichuan, China

**Keywords:** Basic Life Support, CPR training, healthcare professionals, hospital levels, knowledge-based competency, logistic regression

## Abstract

**Background:**

Timely and effective Basic Life Support (BLS) is essential for improving survival after cardiac arrest. However, knowledge-based BLS competency among healthcare professionals (HCPs) working at different hospital levels has not been sufficiently characterized. This multicenter cross-sectional study aimed to assess knowledge-based BLS competency among HCPs and identify factors associated with high knowledge-based BLS competency.

**Methods:**

From March 2025 to March 2026, a cross-sectional survey of 2,017 HCPs (physicians, nurses, technicians, and other clinical staffs) was carried out in tertiary, secondary, and primary hospitals of Chengdu, Sichuan Province in urban areas. Demographic and professional characteristics, CPR training history, and BLS knowledge were assessed using an electronic questionnaire developed by the research team with reference to international BLS guidelines. The internal consistency of the questionnaire in the analytical sample was acceptable, with a Cronbach’s alpha of 0.701. Descriptive statistics, chi-square tests, and multivariable regression analyzes were used to identify factors associated with knowledge-based BLS competency. A total BLS knowledge score of ≥80 points was classified as high knowledge-based BLS competency. Item-level accuracy and domain-specific knowledge scores were also calculated.

**Results:**

Overall, 40.26% of participants met the criterion for high knowledge-based BLS competency. In the unadjusted analyzes, professional role, hospital level, and educational level were significantly associated with high knowledge-based BLS competency. In the multivariable logistic regression model, physicians (OR = 3.01, 95% CI: 1.58–5.74) and nurses (OR = 2.46, 95% CI: 1.31–4.63) had significantly higher odds of achieving high knowledge-based BLS competency than technicians and other clinical staff. HCPs working in tertiary hospitals also had higher odds of achieving high knowledge-based BLS competency than those working in primary hospitals (OR = 1.48, 95% CI: 1.09–2.00). Core CPR knowledge had the highest domain-specific accuracy (87.43%), whereas defibrillation-related knowledge had the lowest accuracy (59.06%).

**Conclusion:**

Knowledge-based BLS competency among the surveyed HCPs working in the participating hospitals remained suboptimal, with particularly notable knowledge gaps in defibrillation-related topics. Professional role, hospital level, and educational background were associated with knowledge-based BLS competency. These findings support the development of standardized, regular, and targeted CPR education, particularly for lower-level hospitals and professional groups with relatively lower knowledge-based BLS competency.

## 1 Introduction

Cardiac arrest is an important cause of death worldwide, and timely implementation of Basic Life Support (BLS) is essential for improving survival following cardiac arrest (1). BLS includes key early interventions such as cardiopulmonary resuscitation (CPR), defibrillation, and airway management, which are particularly important during the first few minutes after cardiac arrest (2). Previous research has demonstrated that timely and high-quality BLS is associated with improved survival outcomes (3). However, gaps in BLS knowledge remain among healthcare professionals (HCPs), and knowledge-based BLS competency may differ across hospital settings. Knowledge-based BLS competency has been less extensively studied in secondary and primary hospitals, which may have different levels of infrastructure, educational resources, and access to structured training compared with tertiary hospitals. These potential differences underscore the need for a cross-institutional assessment of knowledge-based BLS competency.

BLS training for HCPs working at different hospital levels may vary in frequency, content, and quality, which may influence their retention of BLS knowledge and preparedness for cardiac emergencies (4). Tertiary hospitals may provide more extensive educational resources, structured training programs, multidisciplinary clinical environments, and exposure to critically ill patients, all of which may support higher knowledge-based BLS competency. In contrast, HCPs working in secondary and primary hospitals may have less frequent access to training, educational materials, and institutional support. Differences in patient volume, case severity, and exposure to emergency events may also contribute to variation in BLS knowledge. Understanding these differences is important for developing targeted BLS education strategies, particularly in hospitals with relatively limited training resources.

Although previous studies have evaluated BLS knowledge and competency in specific healthcare settings, many have been limited to a single institution, a relatively small sample, or one professional group (5). These studies have identified knowledge gaps in areas such as defibrillation, chest-compression quality, and airway management (6, 7). However, few studies have directly compared knowledge-based BLS competency across tertiary, secondary, and primary hospitals while including multiple categories of HCPs. In addition, previous studies have frequently focused on total knowledge scores without identifying weaknesses at the item or knowledge-domain level. Although participation in BLS training is common, training history and theoretical knowledge do not necessarily reflect psychomotor skills or actual clinical resuscitation performance. A detailed evaluation of knowledge-based BLS competency across hospital settings and professional groups is therefore needed to identify specific educational priorities.

This multicenter cross-sectional study was conducted to assess knowledge-based BLS competency among HCPs working at different hospital levels. The study also sought to identify factors associated with high knowledge-based BLS competency by examining professional role, hospital level, educational background, and CPR training and assessment patterns. The study included HCPs from tertiary, secondary, and primary hospitals and evaluated knowledge-based BLS competency using four complementary approaches: overall competency classification, item-level accuracy, domain-specific knowledge scores, and multivariable regression analysis. This cross-level approach allowed potential knowledge differences associated with individual professional characteristics and institutional context to be examined. The findings provide evidence regarding specific BLS knowledge gaps and may help identify priorities for standardized and targeted education, particularly in lower-level hospitals and professional groups with relatively lower knowledge-based BLS competency.

## 2 Materials and methods

### 2.1 Study design and setting

A multicenter cross-sectional survey was conducted from March 2025 to March 2026 in Chengdu, Sichuan Province among healthcare professionals working in participating tertiary, secondary, and primary hospitals in an urban area. A non-probability voluntary-response sampling approach was used. The participating hospital levels represented healthcare settings with different institutional resources, training opportunities, and emergency-care capacities. The Institutional Review Board (IRB) of the West China Tianfu Hospital, Sichuan University (Approval No. 2024-089) gave ethical approval, and informed consent was gained from all participants prior to their involvement. The purpose of this study was to assess knowledge-based BLS competency and identify factors associated with higher knowledge-based BLS competency across healthcare settings, with particular attention to knowledge of CPR, defibrillation, airway management, and resuscitation quality-control principles.

### 2.2 Participants

The study population comprised physicians, nurses, technicians, and other healthcare professionals with direct clinical or patient-care responsibilities in the participating hospitals. The electronic questionnaire was disseminated through the participating hospitals and professional communication channels, and eligible healthcare professionals voluntarily participated in the survey. Hospital level and professional role were recorded to characterize the respondent sample and were subsequently included as explanatory variables in the statistical analyzes. For statistical analyzes, occupation was categorized into four groups: physician, nurse, technician, and other clinical staff. The category of “other clinical staff” comprised licensed HCPs with direct patient-care responsibilities who were not physicians, nurses, or technicians. Education was grouped as college or lower, bachelor’s and master’s or higher. Professional title was divided into junior, intermediate and senior title. Demographic and professional factors were summarized as baseline characteristics and were considered candidate variables for the subsequent multivariable analyzes.

**Figure 1:**
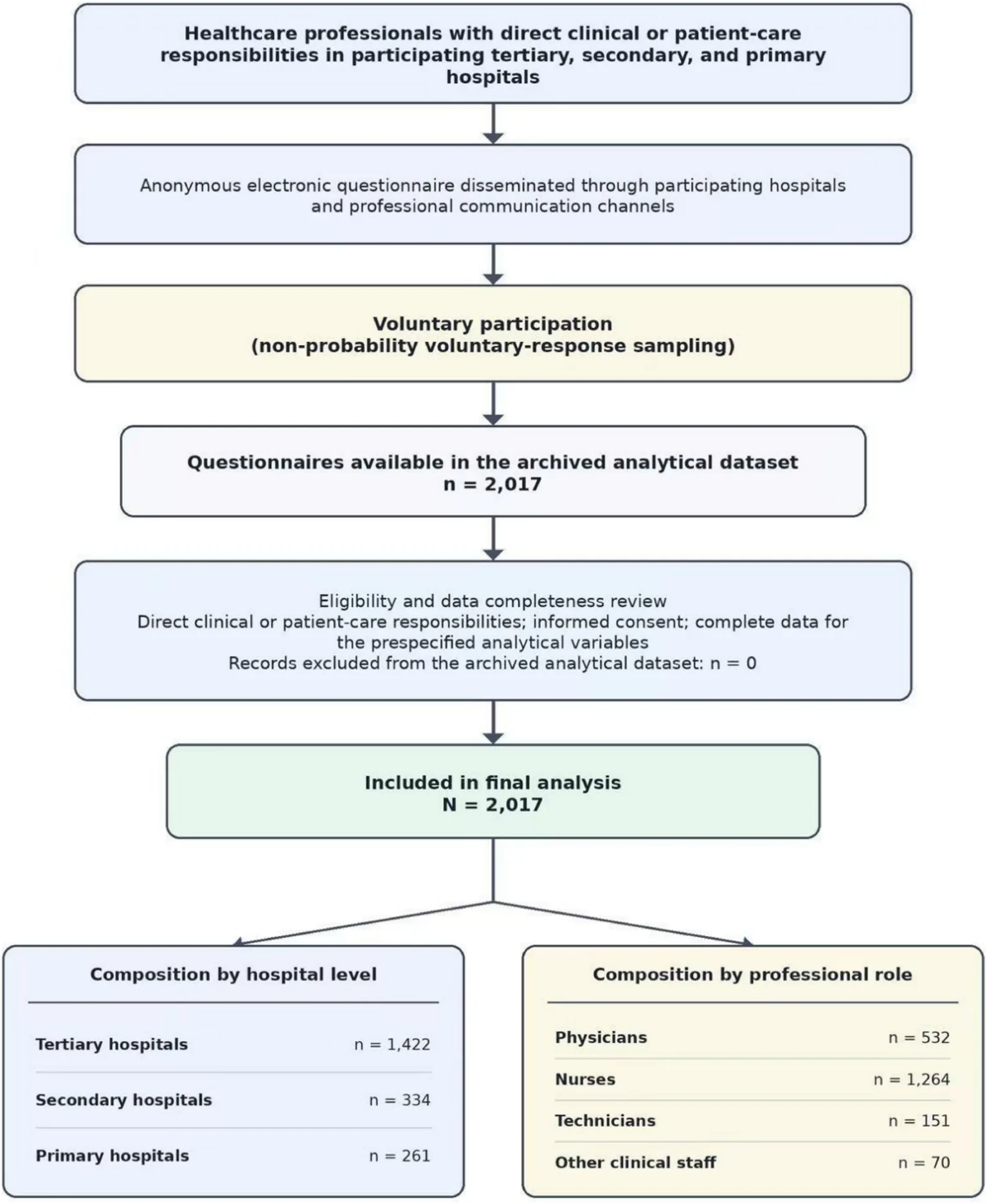
Participant recruitment, inclusion, and sample composition.

Eligible participants were physicians, nurses, technicians, and other healthcare professionals who worked in the participating hospitals, had direct clinical or patient-care responsibilities, voluntarily agreed to participate, provided informed consent, and completed the electronic questionnaire. Non-clinical administrative and support personnel were not eligible for inclusion. The questionnaire was disseminated electronically through the participating hospitals and professional communication channels, and eligible healthcare professionals voluntarily accessed and completed the survey. At the end of the prespecified survey period, the archived analytical dataset contained 2,017 completed questionnaires with the variables required for the planned analyzes. All 2,017 completed questionnaires were included in the final statistical analysis. The participant recruitment, inclusion process, and sample composition are presented in Figure 1.

### 2.3 Sample size calculation

The minimum required sample size was estimated using the formula for a single population proportion. Assuming an expected proportion of 50%, a 95% confidence level, a margin of error of 3%, and *Z* = 1.96, the minimum required sample size was calculated as 1,067. This value represented the minimum number of respondents required to achieve the prespecified level of statistical precision rather than a maximum recruitment limit. Data collection remained open throughout the prespecified survey period, and all 2,017 completed questionnaires available at the end of the survey period were retained for analysis. The final sample exceeded the minimum requirement and provided greater precision for the descriptive estimates and improved stability for the planned multivariable and subgroup analyzes.

### 2.4 Data collection

The data were gathered via a secure online survey platform, with an anonymous electronic questionnaire. The questionnaire included three sections: demographic/professional characteristics, CPR training history and BLS knowledge assessment. Demographic and professional data, such as sex, professional role, work experience, hospital level, educational level, professional title and other professional data were collected in the first section. Educational level was divided into college or less, bachelor’s and master’s or more, and professional title was divided into junior, intermediate and senior title for consistency with baseline and regression results. Work experience was divided into 1–5 years, 6–10 years, 11–20 years and >21 years and the available career-stage indicator was presented in the baseline characteristics table. The second section queried participants pertaining to their prior CPR training, including the number of times they had previously received BLS/CPR training, including within their own hospitals, online, and at academic symposia; interval since last training; and interval since last formal competency assessment. The third section was a 20 questions online knowledge test on the important aspects of BLS such as CPR, defibrillator and airway management. Demographic, professional and training-related variables were prespecified as potential explanatory variables for the subsequent multivariable regression analyzes. The questionnaire was developed by the research team with reference to the 2020 American Heart Association Guidelines for Cardiopulmonary Resuscitation and Emergency Cardiovascular Care and the 2021 European Resuscitation Council Guidelines for Basic Life Support, which were available during questionnaire development and survey administration (8, 9).

The questionnaire was developed by the research team with reference to the American Heart Association Guidelines for Cardiopulmonary Resuscitation and Emergency Cardiovascular Care and the European Resuscitation Council Guidelines for Basic Life Support (8, 9). The questionnaire covered major BLS knowledge domains, including core CPR procedures, airway management, defibrillation-related knowledge, and resuscitation quality-control principles. Before formal distribution, the research team reviewed the questionnaire content, wording, response options, scoring rules, and electronic presentation to improve clarity, readability, and consistency with the source guidelines. This procedure represented an internal review by the research team rather than a formal external expert-validation or Delphi process. No independent pilot study, Content Validity Index, Content Validity Ratio, or other quantitative content-validity assessment was conducted. The final questionnaire comprised 20 knowledge items, and the wording of these items is provided in Supplementary Table 1.

The knowledge-based BLS competency score was computed using the number of correct responses. A point score was assigned to each correct answer (5 points) and each incorrect answer (0 points), to give a total score of between 0 and 100. High knowledge-based BLS competency was determined as a score of ≥80 points, which included a minimum of 16 correct responses out of 20 items. This cut-off was chosen because 80% is a commonly used mastery criteria in health professional training and assessment of knowledge, and represents a reasonably high level of theoretical knowledge of key BLS concepts. Participants who scored <80 points were considered to have lower knowledge-based BLS competency. In this study, knowledge-based BLS competency was assessed using participants’ responses to the questionnaire; hands-on CPR skills, simulation-based performance, and actual clinical resuscitation performance were not assessed.

The online survey platform automatically recorded submitted responses and exported the data to a password-protected database accessible only to authorized members of the research team. Before analysis, the dataset was reviewed for completeness of the prespecified variables, allowable response categories, and internal consistency. The final analytical dataset comprised 2,017 completed questionnaires, all of which were included in the statistical analyzes. After de-identification, the dataset was securely stored and used exclusively for the present study.

### 2.5 Statistical analysis

The total BLS knowledge score was analyzed using multivariable linear regression to identify associated factors. Multivariable logistic regression was used to estimate odds ratios (ORs) and 95% confidence intervals (CIs) for high versus lower knowledge-based BLS competency. Professional role, hospital level, educational level, professional title, CPR training interval, assessment interval, and other clinically relevant demographic variables were considered as candidate explanatory variables. Variables retained in the final multivariable models are presented in Tables 1, 2. Categorical variables were dummy-coded. The reference categories were other clinical staff for professional role, intermediate title for professional title, secondary technical school education for educational level, and primary hospital for hospital level. Dummy variables for all non-reference categories were entered into the corresponding models; only the principal statistically significant associations are presented in Tables 1, 2.

**Table 1:** Multivariable linear regression for total BLS knowledge score.

Variable	Unstandardized *B*	95% CI	*p*
Physician	18.46	14.76–22.17	<0.001
Nurse	16.89	13.30–20.48	<0.001
Junior title	1.51	0.13–2.89	0.032
Senior title	−3.10	−5.39 to −0.80	0.008
Bachelor’s degree	9.64	4.99–14.30	<0.001
Master’s degree or above	7.86	2.68–13.03	0.003
Tertiary hospital	3.75	1.76–5.75	<0.001
Secondary hospital	3.25	0.92–5.58	0.006
Model *R*^2^	0.156		

Results are presented as unstandardized *B* coefficients with 95% CIs. Intermediate title, college or below, primary hospital, and technician/other clinical staff served as the reference categories. Positive *B* values indicate higher adjusted total BLS knowledge scores compared with the corresponding reference category. BLS, Basic Life Support; CI, confidence interval; *R*^2^, coefficient of determination.

**Table 2:** Multivariable logistic regression for high knowledge-based BLS competency (≥80 points).

Variable	Adjusted OR	95% CI	*p*
Physician	3.01	1.58–5.74	<0.001
Nurse	2.46	1.31–4.63	0.005
Tertiary hospital	1.48	1.09–2.00	0.011
Secondary hospital	1.27	0.89–1.81	0.179

Results are presented as adjusted ORs with 95% CIs. Technician/other clinical staff and primary hospital served as the reference categories. OR > 1 indicates higher odds of achieving high knowledge-based BLS competency compared with the corresponding reference category. BLS, Basic Life Support; CI, confidence interval; OR, odds ratio.

Linear regression results were reported as unstandardized *B* coefficients with 95% CIs and *p* values, representing adjusted mean differences in the total BLS knowledge score relative to the corresponding reference category. Logistic regression results were reported as adjusted ORs with 95% CIs and *p* values for the odds of achieving high knowledge-based BLS competency. Variance inflation factors (VIFs) were used to assess multicollinearity, with values >10 considered indicative of potentially important multicollinearity. For the linear regression model, residual plots were examined to assess linearity, homoscedasticity, and approximate normality of residuals. Cook’s distance was calculated to assess the potential influence of individual observations in both regression models, with a value of 1 used as the prespecified screening threshold for substantial influence. The goodness of fit of the logistic regression model was evaluated using the Hosmer–Lemeshow test, with participants grouped into deciles according to their predicted probabilities. The test statistic, degrees of freedom, and *p*-value were reported. A *p* value <0.05 was considered statistically significant.

### 2.6 Internal consistency

Cronbach’s alpha was used to assess the internal consistency of the final 20-item knowledge assessment. The coefficient was 0.701, indicating acceptable internal consistency among the questionnaire items in the present analytical sample. The questionnaire assessed knowledge of core CPR procedures, airway management, defibrillation, and resuscitation quality-control principles.

### 2.7 Ethical considerations

The study was conducted in accordance with the ethical principles of respect for persons, beneficence, and justice. Before completing the questionnaire, participants were informed of the study purpose, the voluntary nature of participation, the protection of confidentiality, and their right to withdraw. Informed consent was obtained from all participants before their involvement. The collected data were anonymized, securely stored, and reported only in aggregate form to protect participant privacy. The study was conducted in accordance with the ethical requirements of the Ethics Committee of West China Tianfu Hospital, Sichuan University (Approval No. 2024-089).

## 3 Results

### 3.1 Basic characteristics and associations with high knowledge-based BLS competency

A total of 2,017 HCPs were included in the final analysis. Most respondents were female (78.48%) and nurses (62.67%). Respondents from tertiary hospitals accounted for 70.50% of the analytical sample, followed by respondents from secondary hospitals (16.56%) and primary hospitals (12.94%). Most participants held a bachelor’s degree (70.75%), while 19.78% had a college-level education or below and 9.47% held a master’s degree or above. Junior professional titles accounted for 49.73% of the sample, followed by intermediate titles (40.56%) and senior titles (9.72%). Regarding work experience, 33.47% had worked for 11–20 years, 28.51% for 1–5 years, 24.69% for 6–10 years, and 13.34% for ≥ 21 years. Hospital level was included in the subsequent multivariable regression models to account for its potential association with the study outcome. Overall, 40.26% of participants met the criterion for high knowledge-based BLS competency, defined as a total BLS knowledge score of ≥80 points.

Unadjusted associations between baseline characteristics and high knowledge-based BLS competency were examined using Pearson’s chi-square tests. Physicians had a higher proportion of high knowledge-based BLS competency (45.30%) than nurses (42.48%), technicians (13.91%), and other clinical staff (18.57%) (*p* < 0.001). The proportion was also higher among HCPs working in tertiary hospitals (42.33%) than among those working in secondary hospitals (39.22%) or primary hospitals (30.27%) (*p* = 0.001). Educational level was significantly associated with high knowledge-based BLS competency (*p* = 0.005), with the highest proportion observed among participants holding a bachelor’s degree (42.47%). Professional title, years of work experience, and sex were not significantly associated with high knowledge-based BLS competency (*p* = 0.278, *p* = 0.104, and *p* = 0.602, respectively). Because respondents from tertiary hospitals were overrepresented, the unadjusted comparisons by hospital level should be interpreted as descriptive rather than as definitive evidence of institutional differences. These results indicate that professional role, hospital level, and educational level might be related to knowledge-based BLS competency as illustrated in Table 3.

**Table 3:** Baseline characteristics and unadjusted associations with high knowledge-based BLS competency (≥80 points).

Variable	Category	*n* (%)	High knowledge-based BLS competency, *n* (%)	Chi-square	*p*
Sex	Female	1,583 (78.48)	642 (40.56)	0.27	0.602
	Male	434 (21.52)	170 (39.17)		
Occupation	Physician	532 (26.38)	241 (45.30)	65.51	<0.001
	Nurse	1,264 (62.67)	537 (42.48)		
	Technician	151 (7.49)	21 (13.91)		
	Other	70 (3.47)	13 (18.57)		
Hospital level	Tertiary	1,422 (70.50)	602 (42.33)	13.53	0.001
	Secondary	334 (16.56)	131 (39.22)		
	Primary	261 (12.94)	79 (30.27)		
Educational level	College or below	399 (19.78)	134 (33.58)	10.81	0.005
	Bachelor’s degree	1,427 (70.75)	606 (42.47)		
	Master’s degree or above	191 (9.47)	72 (37.70)		
Professional title	Junior title	1,003 (49.73)	418 (41.67)	2.56	0.278
	Intermediate title	818 (40.56)	312 (38.14)		
	Senior title	196 (9.72)	82 (41.84)		
Years of experience	1–5 years	575 (28.51)	232 (40.35)	6.16	0.104
	6–10 years	498 (24.69)	219 (43.98)		
	11–20 years	675 (33.47)	267 (39.56)		
	≥21 years	269 (13.34)	94 (34.94)		

High knowledge-based BLS competency was defined as a total BLS knowledge score of ≥80 points. Data are presented as *n* (%). Percentages in the “High knowledge-based competency, *n* (%)” column represent the proportion of participants meeting the criterion within each category. Group differences were assessed using Pearson’s chi-square test. Chi-square and *p* values are shown only in the first row of each variable. BLS, Basic Life Support.

### 3.2 CPR training and assessment patterns

Most participants (96.58%) reported having access to CPR training materials. Regarding the interval since the most recent CPR/BLS training, 39.76% had received training within 3 months, 28.85% within 3–6 months, and 24.59% within 6–12 months. Regarding formal CPR/BLS assessment, 34.36% had been assessed within 3 months, whereas 2.83% reported that they had never undergone a formal assessment. These findings describe the distribution of self-reported training and assessment intervals among the surveyed participants and are summarized in Table 4.

**Table 4:** CPR training and assessment patterns.

Variable	Category	*n* (%)
Training materials	Yes	1,948 (96.58)
	No	69 (3.42)
Training interval	≤3 months	802 (39.76)
	3–6 months	582 (28.85)
	6–12 months	496 (24.59)
	1–2 years	105 (5.21)
	Never trained	32 (1.59)
Assessment interval	≤3 months	693 (34.36)
	3–6 months	601 (29.80)
	6–12 months	535 (26.52)
	≥1 year	131 (6.49)
	Never assessed	57 (2.83)

Data are presented as *n* (%). Training interval refers to the interval since the most recent CPR/BLS training. Assessment interval refers to the interval since the most recent formal CPR/BLS knowledge assessment. BLS, Basic Life Support; CPR, cardiopulmonary resuscitation.

### 3.3 Item-level accuracy of BLS knowledge

**Figure 2:**
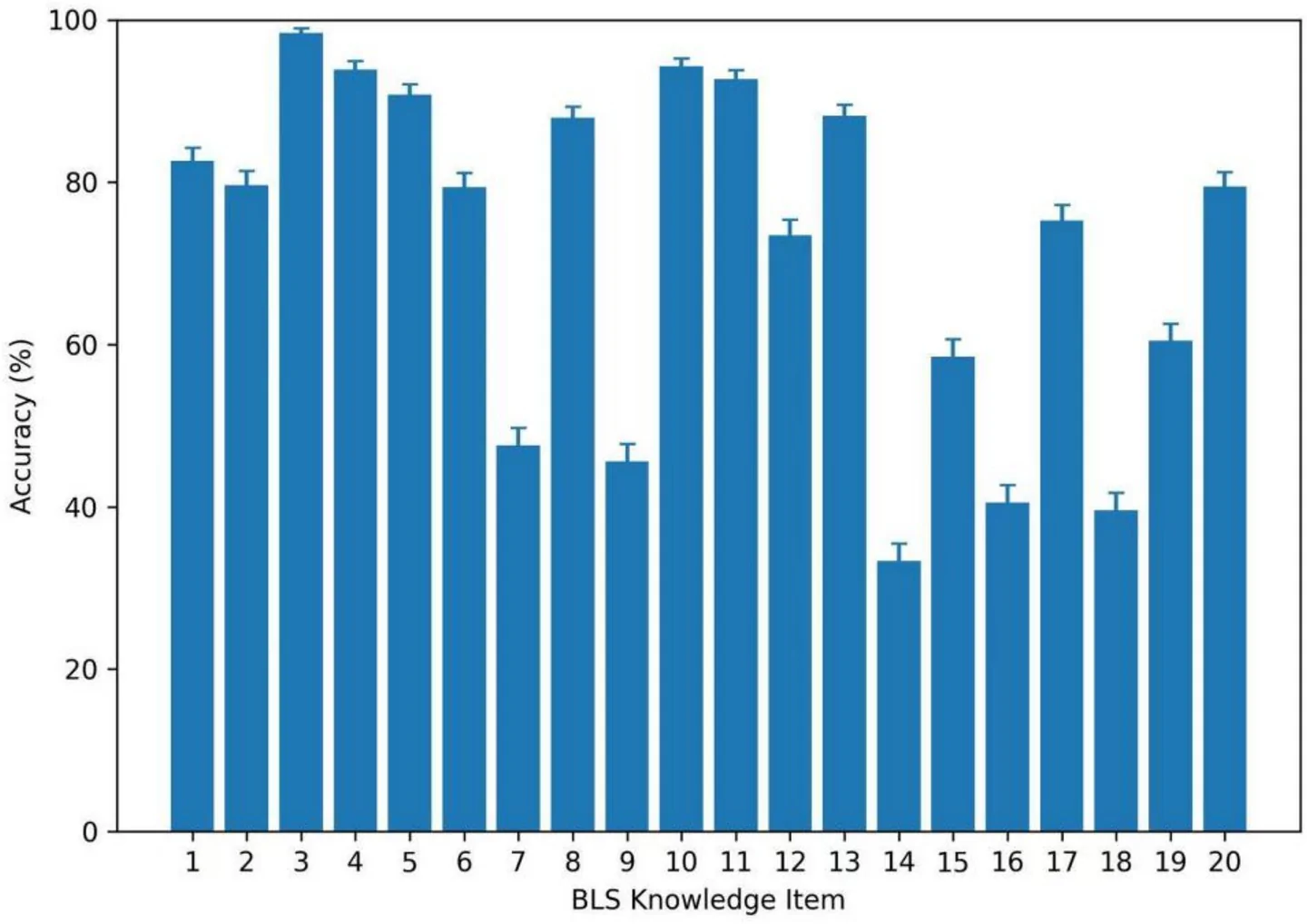
Item-level accuracy of the knowledge-based BLS competency assessment among healthcare professionals (*N* = 2,017), with 95% confidence intervals.

Item-level accuracy varied considerably across the 20 BLS knowledge questions. The highest accuracy was observed for Q3 (98.36%), followed by Q10 (94.25%), Q4 (93.85%), and Q11 (92.71%). In contrast, the lowest accuracy was observed for Q14 (33.37%), followed by Q18 (39.56%), Q16 (40.51%), and Q9 (45.56%). These results indicate specific knowledge gaps in airway-management, defibrillation-related, and resuscitation quality-control topics. Item-level accuracy with 95% CIs is presented in Table 5 and Figure 2.

**Table 5:** Item-level accuracy with 95% CIs.

Item	Correct *n*	Accuracy (%)	95% CI
Q1	1,666	82.60	80.91–84.23
Q2	1,606	79.62	77.84–81.36
Q3	1,984	98.36	97.77–98.91
Q4	1,893	93.85	92.76–94.89
Q5	1,831	90.78	89.49–92.02
Q6	1,601	79.38	77.59–81.11
Q7	959	47.55	45.36–49.73
Q8	1,773	87.90	86.47–89.29
Q9	919	45.56	43.38–47.74
Q10	1,901	94.25	93.21–95.24
Q11	1,870	92.71	91.57–93.80
Q12	1,481	73.43	71.49–75.36
Q13	1,778	88.15	86.71–89.54
Q14	673	33.37	31.33–35.45
Q15	1,180	58.50	56.37–60.63
Q16	817	40.51	38.37–42.64
Q17	1,519	75.31	73.43–77.19
Q18	798	39.56	37.43–41.70
Q19	1,219	60.44	58.30–62.57
Q20	1,603	79.47	77.69–81.21

CI, confidence interval. Accuracy was calculated as the proportion of participants who answered each item correctly.

### 3.4 Domain-specific knowledge scores

**Figure 3:**
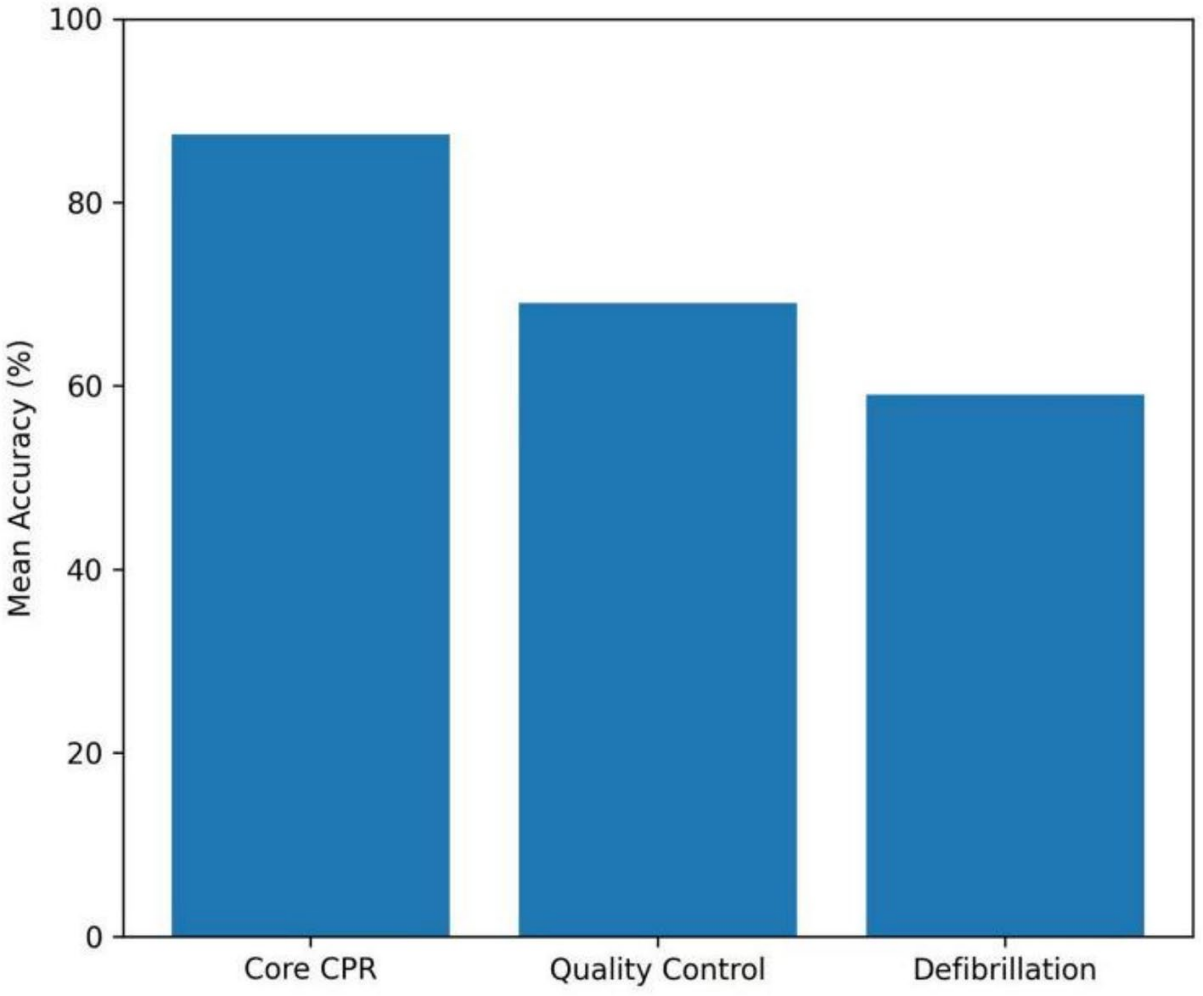
Domain-specific mean accuracy of the knowledge-based BLS competency assessment among healthcare professionals.

Among the three BLS knowledge domains, core CPR procedures had the highest mean accuracy (87.43%), followed by resuscitation quality-control principles (69.05%) and defibrillation-related knowledge (59.06%). These domain-specific findings indicate relatively strong knowledge of core CPR procedures but lower knowledge of defibrillation-related and resuscitation quality-control topics. The overall mean accuracy across the 20 items was 72.06% ± 15.43%. The domain-specific results are summarized in Table 6 and presented graphically in Figure 3.

**Table 6:** Domain-specific knowledge scores.

Domain	Items	Mean accuracy (%)
Core CPR	Q1–Q6	87.43
Quality control	Q7–Q15	69.05
Defibrillation	Q16–Q20	59.06
Overall	Q1–Q20	72.06 ± 15.43

Domain-specific accuracy was calculated by averaging the accuracy rates of items within each domain. CPR, cardiopulmonary resuscitation.

### 3.5 Multivariable linear regression for total BLS knowledge score

Multivariable linear regression was done to determine the factors associated with the total BLS knowledge score. The distribution of the sample was weighted toward tertiary hospitals and it was felt that the level of hospital needed to be accounted for in the regression model due to its potential impact on the total BLS knowledge score. Results are presented as mean differences in total BLS knowledge score relative to the appropriate reference group (unstandardized *B* coefficients). Physicians and nurses had significantly higher BLS scores than the reference group, with *B* coefficients of 18.46 (95% CI: 14.76–22.17, *p* < 0.001) and 16.89 (95% CI: 13.30–20.48, *p* < 0.001), respectively. Educational level was also significantly associated with total BLS knowledge score, with higher scores observed among participants with a bachelor’s degree (*B* = 9.64, 95% CI: 4.99–14.30, *p* < 0.001) or a master’s degree (*B* = 7.86, 95% CI: 2.68–13.03, *p* = 0.003). After adjustment, working in a tertiary hospital (*B* = 3.75, 95% CI: 1.76–5.75, *p* < 0.001) or secondary hospital (*B* = 3.25, 95% CI: 0.92–5.58, *p* = 0.006) remained positively associated with total BLS knowledge score. Table 1 shows that the model accounted for 15.63% of the variance in the total BLS knowledge scores (*R*^2^ = 0.156). The maximum VIF value was 11.65, exceeding the prespecified threshold of 10. The maximum Cook’s distance was 0.068, which was below the prespecified screening threshold of 1, suggesting that no single observation exerted substantial influence on the model estimates. Examination of the residual plots did not reveal substantial departures from linearity, homoscedasticity, or approximate normality.

### 3.6 Predictors of high knowledge-based BLS competency: logistic regression

**Figure 4:**
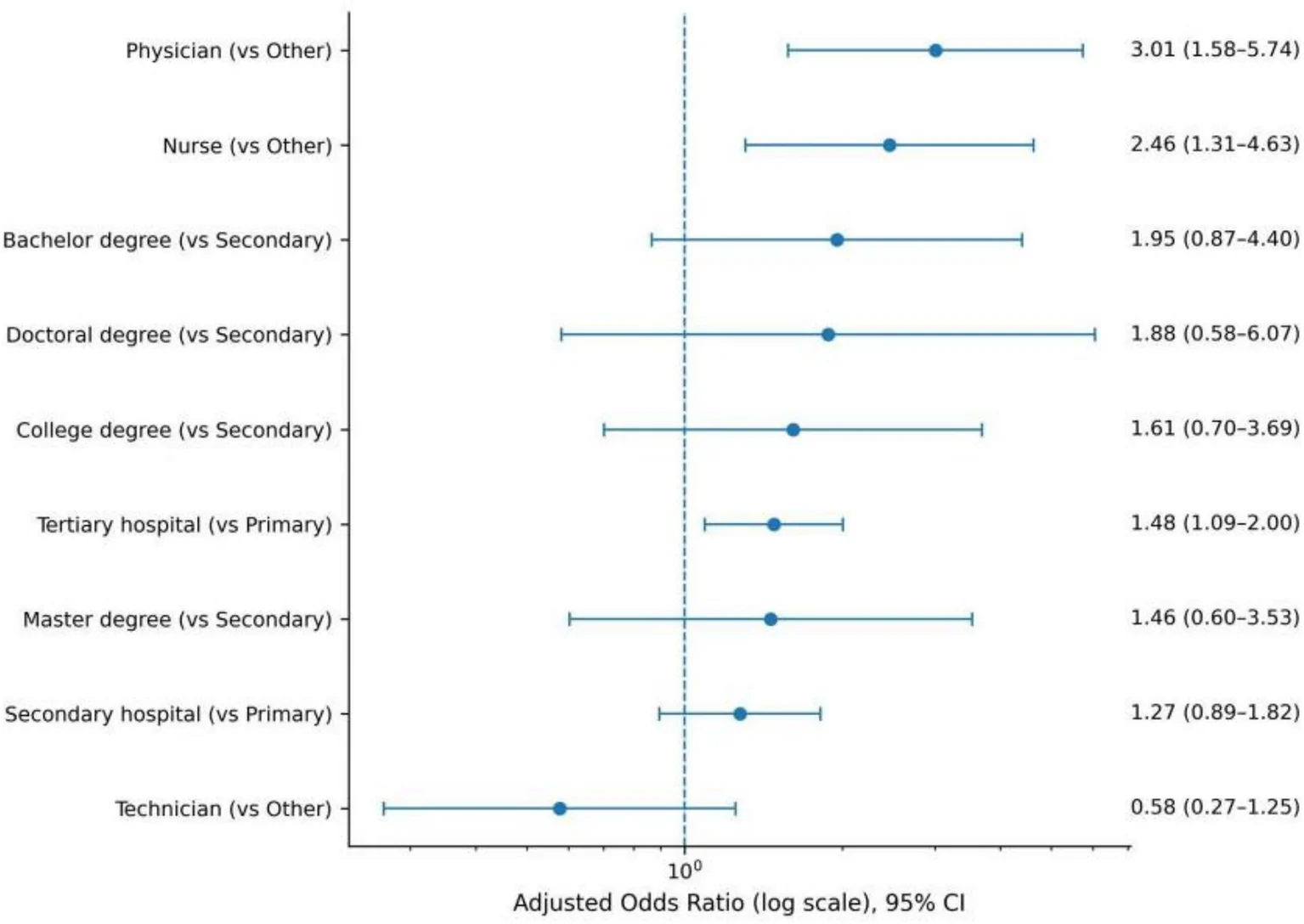
Adjusted odds ratios for factors associated with high knowledge-based BLS competency, defined as a total BLS knowledge score of ≥80 points.

Multivariable logistic regression was performed to identify factors associated with high knowledge-based BLS competency, defined as a total BLS knowledge score of ≥80 points. Because respondents from tertiary hospitals represented a relatively large proportion of the analytical sample, hospital level was included as an explanatory variable to estimate its association with high knowledge-based BLS competency after adjustment for other covariates. Results are presented as adjusted ORs with 95% CIs, with technicians/other clinical staff serving as the reference category for professional role and primary hospitals serving as the reference category for hospital level. Physicians (OR = 3.01, 95% CI: 1.58–5.74, *p* < 0.001) and nurses (OR = 2.46, 95% CI: 1.31–4.63, *p* = 0.005) had significantly higher odds of achieving high knowledge-based BLS competency than the reference professional group. HCPs working in tertiary hospitals also had higher odds of achieving high knowledge-based BLS competency than those working in primary hospitals (OR = 1.48, 95% CI: 1.09–2.00, *p* = 0.011). The association for secondary hospitals was not statistically significant (OR = 1.27, 95% CI: 0.89–1.81, *p* = 0.179). These results suggest that hospital level was associated with knowledge-based BLS competency after adjustment, although the overrepresentation of tertiary-hospital respondents should be considered when interpreting the magnitude and generalizability of this association. The maximum VIF value in the final logistic regression model was 11.59, exceeding the prespecified threshold of 10. The Hosmer–Lemeshow goodness-of-fit test yielded *χ*^2^(8) = 10.90, *p* = 0.208, providing no statistically significant evidence of poor model fit. The maximum Cook’s distance was 0.021, which was below the prespecified screening threshold of 1 and suggested that no single observation exerted substantial influence on the model estimates. The results are summarized in Table 2 and presented graphically in Figure 4.

## 4 Discussion

This multicenter cross-sectional study assessed knowledge-based BLS competency among HCPs working at different hospital levels and examined its associations with professional, educational, institutional, and training-related factors. Knowledge-based BLS competency varied across professional roles, hospital levels, educational levels, and training and assessment patterns. Given the use of voluntary-response sampling and the predominance of respondents from tertiary hospitals, these findings should be interpreted as characterizing the surveyed HCPs in the participating hospitals rather than HCPs as a whole. Unlike many previous studies that assessed BLS knowledge within a single institution, one professional group, or through an overall score alone, the present study included HCPs from tertiary, secondary, and primary hospitals and used overall classification, item-level accuracy, domain-specific accuracy, and multivariable regression analysis. This multidimensional approach provides additional evidence regarding specific BLS knowledge gaps and factors associated with knowledge-based BLS competency (4, 10). The outcome assessed in this study reflects questionnaire-based knowledge rather than psychomotor BLS skills or actual clinical resuscitation performance. The findings may therefore help identify priorities for standardized and targeted BLS education, particularly in lower-level hospitals and professional groups with relatively lower knowledge-based BLS competency.

Professional role was significantly associated with knowledge-based BLS competency. Physicians and nurses had higher total BLS knowledge scores and higher odds of achieving high knowledge-based BLS competency than technicians and other clinical staff. This finding is consistent with previous evidence suggesting that physicians may have greater exposure to emergency situations and more frequent access to advanced or structured training programs (10, 11). Nurses also play a central role in recognizing clinical deterioration and initiating early responses during in-hospital cardiac arrest. These findings support the development of BLS education that is broadly accessible and adapted to the responsibilities and identified knowledge gaps of different professional groups. Bushuven et al. (12) similarly reported variation in CPR-related knowledge, confidence, and training experiences across professional groups. Differences in clinical exposure and access to repeated training may partly explain the observed variation, although this interpretation requires confirmation in future studies.

Hospital level was associated with knowledge-based BLS competency (13). Compared with respondents working in primary hospitals, those working in tertiary hospitals had higher adjusted total BLS knowledge scores and higher odds of achieving high knowledge-based BLS competency. This difference may reflect variation in training opportunities, educational resources, simulation capacity, and exposure to critically ill patients. Wang et al. (13) similarly reported differences in first-aid capabilities and training needs across hospital settings. Nevertheless, hospital level should be interpreted as an institutional marker rather than a direct causal mechanism because individual hospitals may differ in their training quality, emergency-response systems, workforce composition, and available resources (4, 13).

Educational background was associated with the total BLS knowledge score (5). Participants with a bachelor’s or master’s degree had higher adjusted knowledge scores than the reference group, possibly reflecting greater exposure to theoretical medical and clinical education (12). However, formal education alone may not ensure effective practical BLS performance, particularly because psychomotor skills were not assessed in the present study. This interpretation is consistent with Gupta et al. (14), who reported that theoretical learning alone may not ensure the effective practical application of BLS techniques. These findings support combining knowledge-based instruction with hands-on and simulation-based training.

Participants reported varying intervals since their most recent CPR/BLS training and formal assessment. Although most respondents had received training and assessment within the preceding 6 months, the present study did not establish an independent or causal association between training recency and knowledge-based BLS competency. Previous research suggests that retraining may support the retention of resuscitation knowledge and skills (15), while resuscitation education guidelines and a comprehensive scoping review support spaced learning, refresher or booster sessions, feedback-informed practice, simulation-based education, and objective performance assessment (16–18). Longitudinal and interventional studies are needed to determine how training frequency, educational format, and hands-on practice influence sustained BLS knowledge and practical performance. In addition, the subsequent 2025 American Heart Association and European Resuscitation Council adult BLS guideline updates should be considered in the future development and revision of BLS knowledge assessments (2, 19).

This study has several limitations. First, the cross-sectional design precludes causal inference; therefore, the factors identified in this study should be interpreted as associated factors rather than causal determinants of knowledge-based BLS competency. Second, information regarding previous training and assessment patterns was self-reported and may have been affected by recall or reporting bias. Third, the use of non-probability voluntary-response sampling may have introduced self-selection bias, as healthcare professionals with a greater interest in BLS, CPR training, or emergency care may have been more likely to participate. Accordingly, the numbers of individuals who viewed the invitation but did not participate or who began but did not submit the questionnaire could not be separately quantified. In addition, 70.50% of respondents worked in tertiary hospitals, and all participants were recruited from hospital-based settings; therefore, the findings primarily reflect the HCPs who participated in the survey, particularly those working in the participating tertiary hospitals, and should be generalized cautiously to HCPs working in other institutions, regions, primary-care settings, prehospital emergency services, or other healthcare environments. Fourth, knowledge-based BLS competency was assessed using a knowledge-based questionnaire rather than direct observation of psychomotor CPR skills or performance in actual resuscitation situations. Accordingly, the findings should be interpreted as reflecting knowledge-based BLS competency rather than overall clinical resuscitation competence. Fifth, the prespecified threshold of ≥ 80 points, corresponding to at least 16 correct responses among the 20 knowledge items, was used as an operational and mastery-oriented criterion for classifying high knowledge-based BLS competency; however, this cutoff should not be regarded as a comprehensive measure of clinical competence. In addition, the maximum VIF values exceeded 10 in both multivariable models, indicating potential multicollinearity that may have reduced the precision and stability of individual coefficient estimates. Finally, although the questionnaire assessed core BLS knowledge, airway management, defibrillation-related knowledge, and resuscitation quality-control principles, a knowledge assessment alone cannot fully capture the multidimensional nature of overall clinical BLS competency. Although the questionnaire was developed with reference to internationally recognized BLS guidelines, Cronbach’s alpha assessed internal consistency only and did not establish the overall validity of the questionnaire. The questionnaire did not undergo formal external content validation or independent pilot testing, and no Content Validity Index, Content Validity Ratio, or other quantitative content-validity measure was calculated. Further external validation and psychometric evaluation in independent samples are therefore warranted. Future studies should incorporate objective skills assessments, simulation-based performance measures, and broader sampling strategies to provide a more comprehensive evaluation of knowledge-based BLS competency.

Future longitudinal studies should include objective skills assessments, simulation-based assessments, or observations of clinical performance to determine whether repeated BLS training improves sustained BLS knowledge, practical performance, or clinical outcomes. Additional further studies should also be conducted on the impediments to frequent trainings and assessments, especially the lower hospitals. These studies may clarify how BLS knowledge can be translated into effective resuscitation performance and emergency preparedness. In particular, links between structured knowledge examinations and standardized practical examinations could lead to a more complete assessment of knowledge-based BLS competency in future research.

Finally, this multicenter study revealed that knowledge-based BLS competency among the surveyed HCPs working in the participating hospitals remained suboptimal and differed according to professional role and hospital level. The results also identified specific knowledge gaps in defibrillation-related and resuscitation quality-control topics. By combining overall competency classification, item-level accuracy, domain-specific scores, and multivariable analyzes, this study provides evidence that may inform BLS educational priorities within the participating hospital settings. Targeted education may be particularly relevant for respondents from secondary and primary hospitals and for professional groups with relatively lower knowledge-based BLS competency. In future training, regular refresher training, periodic assessment, simulation based education and training, and more strategic allocation of training resources may be considered; however additional studies (longitudinal or interventional) should be conducted to assess their impact on knowledge-based BLS competency and patient safety. Future studies incorporating objective performance measures and broader, more balanced samples would complement the present cross-sectional, questionnaire-based findings and support the further optimization of BLS training programs.

## Data Availability

The raw data supporting the conclusions of this article will be made available by the authors, without undue reservation.
