# Supplementary material for: Knowledge-based Basic Life Support competency and associated factors among healthcare professionals across different hospital levels: a multicenter cross-sectional study
Source: Front Public Health. 2026 Aug 20;14:1881120. doi: 10.3389/fpubh.2026.1881120 (PMC13540587; doi:10.3389/fpubh.2026.1881120)
Supplement: Supplementary file 1 [file Supplementary_file_1.docx]

Questionnaire on CPR Training and Basic Life Support Knowledge

| Respondent ID: ____________________ | Date: ____________________ |
| --- | --- |

Part I. General Information

| **Item** | **Response options** |
| --- | --- |
| **Gender** | □ Male  □ Female |
| **Profession** | □ Physician  □ Nurse  □ Medical technician  □ Other |
| **Years of work experience (years)** | □ 1–5 years  □ 6–10 years  □ 11–20 years  □ ≥21 years |
| **Educational level** | □ Secondary vocational / technical school diploma  □ Junior college / associate degree  □ Bachelor’s degree  □ Master’s degree  □ Doctoral degree |
| **Professional title** | □ Junior  □ Intermediate  □ Senior |
| **Level of your institution** | □ Tertiary hospital  □ Secondary hospital  □ Primary hospital |
| **Total number of out-of-hospital and in-hospital cardiac arrest patients treated in your department each year (cases)** | □ No such patients  □ 1–5  □ 6–10  □ 11–30  □ 31–50  □ ≥51 |

Part II. Previous Training and Assessment

| **Item** | **Response options** |
| --- | --- |
| **Does your department have materials related to CPR theory and skills training?** | □ Yes  □ No |
| **Formats through which you have received CPR training** | □ In-hospital training  □ Internet-based learning  □ Academic conferences  □ Journal articles  □ Other |
| **Interval between CPR theory and skills training sessions received in your department** | □ Within 3 months  □ 3–6 months  □ 6 months to 1 year  □ 1–2 years  □ Never received training |
| **Interval between CPR theory and skills assessments conducted in your department** | □ Within 3 months  □ 3–6 months  □ 6 months to 1 year  □ 1–2 years  □ More than 2 years  □ Never been assessed |

Part III. Basic Life Support Knowledge

1. What is the correct procedure for assessing a patient’s breathing and pulse?

□ Assess breathing and pulse simultaneously, completing the check within 10 seconds

□ Assess breathing and pulse for at least 10 seconds or longer

□ Assess breathing alone for 15–20 seconds

□ Assess the pulse alone for 10–15 seconds

2. During cardiopulmonary resuscitation (CPR), A denotes airway opening, B denotes rescue breathing, and C denotes chest compressions. What is the correct CPR sequence?

| □ A–B–C | □ C–B–A |
| --- | --- |
| □ C–A–B | □ Not sure |

3. During on-site manual CPR, what is the correct position for the patient?

| □ Supine on a comfortable soft bed | □ Supine on a firm, flat surface |
| --- | --- |
| □ Lateral recumbent position | □ Prone position |

4. Where should external chest compressions be delivered during CPR?

| □ Middle of the sternum | □ Midpoint of the line connecting the nipples |
| --- | --- |
| □ Cardiac apex | □ Upper-middle portion of the sternum |

5. What is the target rate for adult chest compressions during CPR?

| □ 100–120 compressions/min | □ Approximately 100 compressions/min |
| --- | --- |
| □ 120–140 compressions/min | □ As fast as possible |

6. What is the target depth for adult chest compressions during CPR?

| □ At least 5 cm but no more than 6 cm | □ 4–5 cm |
| --- | --- |
| □ At least 6 cm | □ As deep as possible |

7. During two-rescuer CPR, how often should the compressor be switched to maintain high-quality chest compressions?

| □ Every 2 minutes | □ Every 3 minutes |
| --- | --- |
| □ Every 4 minutes | □ Every 5 minutes |

8. What is the compression-to-ventilation ratio for two-rescuer adult CPR?

| □ 30:2 | □ 15:2 |
| --- | --- |
| □ 15:1 | □ Continuous compressions; no ventilation |

9. During adult CPR, if compressors need to be switched, each interruption in chest compressions should not exceed:

| □ 5 seconds | □ 10 seconds |
| --- | --- |
| □ 20 seconds | □ 30 seconds |

10. During adult CPR, after how many cycles should the effectiveness of resuscitation be reassessed?

| □ Perform checks and analysis at any time | □ 5 cycles |
| --- | --- |
| □ 4 cycles | □ 6 cycles |

11. What is the most commonly used technique for opening the airway during adult CPR?

| □ Chin-lift maneuver | □ Head-tilt–chin-lift maneuver |
| --- | --- |
| □ Two-handed jaw-thrust maneuver | □ Cricoid pressure |

12. Which airway-opening technique should be used when cervical spine injury is suspected?

| □ Two-handed jaw-thrust maneuver | □ Head-tilt–chin-lift maneuver |
| --- | --- |
| □ Chin-lift maneuver | □ Cricoid pressure |

13. Which one-handed technique is used to secure the mask of a bag-valve-mask (BVM) device?

| □ D–E technique | □ E–C technique |
| --- | --- |
| □ E–B technique | □ B–C technique |

14. During ventilation, over how long should each breath be delivered with a bag-valve-mask (BVM) device?

| □ >1 second | □ 1 second |
| --- | --- |
| □ >2 seconds | □ 3 seconds |

15. After placement of an advanced airway, how often should ventilation be provided while continuous chest compressions are maintained?

□ One breath every 6 seconds (10 breaths/min)

□ One breath every 12 seconds (5 breaths/min)

□ One breath every 10 seconds (6 breaths/min)

□ One breath every 30 seconds (2 breaths/min)

16. Where should the two defibrillator electrodes be placed?

□ Base pad: below the right clavicle at the right sternal border; apex pad: left 5th intercostal space at the midaxillary line

□ Base pad: right midclavicular line at the 2nd–3rd intercostal space; apex pad: left anterior axillary line at the 6th–7th intercostal space

□ Base pad: left midclavicular line at the 2nd–3rd intercostal space; apex pad: left anterior axillary line at the 7th–8th intercostal space

□ Base pad: right midclavicular line at the 2nd–3rd intercostal space; apex pad: right anterior axillary line at the 5th–6th intercostal space

17. What initial energy dose is recommended for defibrillation of a patient with ventricular fibrillation using a monophasic waveform?

| □ 340 J | □ 350 J |
| --- | --- |
| □ 360 J | □ 370 J |

18. What initial energy dose is recommended for defibrillation of a patient with ventricular fibrillation using a biphasic waveform?

| □ 100–200 J | □ 110–200 J |
| --- | --- |
| □ 120–200 J | □ 130–200 J |

19. What should be done immediately after delivery of a defibrillation shock?

| □ Check the cardiac rhythm | □ Resume chest compressions |
| --- | --- |
| □ Check a central pulse | □ Immediately deliver a second shock |

20. Which defibrillation mode should be selected for ventricular fibrillation?

| □ Synchronized | □ Unsynchronized |
| --- | --- |
| □ Synchronized first, then unsynchronized | □ Not sure |
